# Dinner in the dark: Factors influencing leopard activity patterns within a large protected area

**DOI:** 10.1371/journal.pone.0324329

**Published:** 2025-05-22

**Authors:** Lucy K. Smyth, Guy A. Balme, M. Justin O’Riain

**Affiliations:** 1 Institute for Communities and Wildlife in Africa, Department of Biological Sciences, University of Cape Town, Cape Town, South Africa; 2 Panthera, New York, United States of America; Smithsonian Conservation Biology Institute, UNITED STATES OF AMERICA

## Abstract

Spatial avoidance is one of the most pervasive responses of wildlife to human disturbance, but new research is increasingly revealing the importance of temporal shifts in activity. Animal species appear to become more nocturnal in areas with greater levels of human disturbance given the predominantly diurnal nature of anthropogenic activity. Here we investigate the relative importance of anthropogenic (relative abundance of pedestrians only, vehicles only and humans on foot and in vehicles combined, artificial light at night and distance to reserve edge or human settlements) and ecological (relative abundance of potential prey and potential competitors, temperature, season and lunar light) factors on the activity patterns of leopards (*Panthera pardus*) in the greater Kruger National Park (KNP) region of southern Africa. We use independent captures from camera trap data collected across 10 sites in and around central and southern KNP as an index of leopard activity, and kernel density models to estimate activity patterns of leopards. No differences in overall leopard activity levels were evident between sites but activity patterns within a 24-hour period differed. These differences were best explained by the relative abundance of pedestrians, with the relative abundance of all humans (on foot or in a vehicle) affecting timing of leopard activity almost as strongly, followed by the relative abundance of vehicles. Leopard activity also varied significantly with the lunar cycle, peaking around full moon. These results suggest that diel leopard activity patterns in South Africa’s largest protected area are primarily driven by anthropogenic not environmental factors, though moonlight is important on the lunar scale. The sensitivity of leopard activity to nocturnal illumination from the moon urges caution regarding levels of human induced artificial light at night which are increasing globally, and could reach a threshold at which they begin to affect leopard activity patterns.

## Introduction

The activity patterns of animals are driven by a multitude of factors which occur at different scales and are driven by both environmental and biological cues. One of the strongest environmental drivers of activity patterns is light [1]. Light affects activity patterns through seasonal changes in day length, monthly changes in the lunar cycle and daily changes in illumination as the earth rotates around the sun [1]. While seasonal variation increases with latitude, all parts of the world experience lunar cycles and daily variations in light which result in diel activity patterns among virtually all species [2].

While light is undeniably important in regulating the activity patterns of animals [1], different animal species show different activity levels under different levels of light [2]. This results in temporal niche partitioning which promotes the ecological separation of species, allowing a wide variety of species to exist within the same ecosystem [3,4]. Temporal niche partitioning can be seen through the existence of diurnal, nocturnal and crepuscular species, facilitating species coexistence within the same environment and reducing competition [2,5].

The amount of light at night varies due to naturally occurring phenomena such as lunar cycles and cloud cover, which have been found to affect timing of activity across a range of species [4,6,7]. However, while the earth’s light regimes have remained relatively unchanged over geological timescales, the Anthropocene has seen a sudden and novel rise in artificial lighting at night which is increasing by an average of 6% annually and is now classified as a form of pollution [1,8]. The newly emerging threat to ecosystem integrity produced by artificial light at night is concerning because of the huge distances travelled by light [1].

Changes to the earth’s natural lighting regime affect the natural environment at many scales, from changes in the behaviour of individuals through to changes in population dynamics and the structure and composition of communities [8–11]. Activity patterning is intricately linked to timing of food availability, hunting success and concealment and is therefore an important component in predator-prey relationships [9,12,13]. The presence of artificial light at night has the potential to affect an animal’s ability to perceive naturally occurring shifts in light over a 24-hour period, changing their time activity budgets and consequently impacting interspecific relationships [1,8]. In response to artificial light, animals can lengthen or shorten their activity periods, shift peaks in activity to earlier or later or respond spatially by relocating [14–16]. All of these changes will however impact other species with which they come into contact, affecting competitive and predator-prey relationships [9,16–18]. The exponential growth in the presence of artificial light at night is therefore reshaping both ecological and evolutionary processes [19–23].

The effects of varying levels of light, both natural and artificial, across the nighttime landscape differ on a species-specific basis. Responses to changes in moonlight have been found to be closely related to a species’ primary sensory modality, though these do seem to vary with habitat cover [24]. Prey species relying mostly on vision as their primary sensory system tend to increase activity levels at night during periods of increased illumination [24–26]. This response in prey species is likely due to a heightened ability to detect predators in the presence of increased moonlight, resulting in lower vulnerability and swaying the risk reward balance in favour of foraging as opposed to vigilance and has been observed across a wide range of taxa with high levels of visual acuity [24–27]. Predatory species however do not appear to show a consistent pattern of changes in activity relative to lunar illumination, regardless of their reliance on vision [25–28]. Within the suite of African predators, this has been explored most extensively in lions, where conflicting results have been reported, with some studies showing a decrease in activity levels with increased moonlight [29] and others finding no change in activity throughout the lunar cycle [17,24]. While little consensus has been reached regarding the impact of moonlight on lion activity levels, hunting success in lions is consistently reported as being higher at times of lower lunar illumination [7,29,30]. This is presumed to be the result of increased prey vulnerability and leads to the conclusion that lower levels of light lead to greater decreases in predator detection by prey than prey detection by predators. While the impacts of light on leopard (*Panthera pardus*) activity levels and hunting success are less well understood, data from leopards in the Cederberg Mountains of South Africa indicates that they too make more kills during periods of lower light [31]. Additionally, a study in Kenya revealed that leopard activity levels were highest at times of greater moon illumination, potentially to compensate for lower hunting success [28], and leopard activity in northern China has also been found to be highest during periods of increased moonlight [25].

Temperature is also important in the timing of animal activity [32–34]. While temperature extremes are a strong determinant of the species assemblage that can persist in a given region [35], within regions daily temperature variation sets limits on the time periods during which different species can be active [36,37]. As temperatures reach more extreme levels for a particular environment an animal’s ability to thermoregulate, thereby maintaining its body temperature within the required range, decreases and activity is usually reduced [38]. Broadly, larger animals are more constrained by heat dissipation, meaning that they are more impacted by higher temperatures, while smaller animals are more constrained by heat retention, and therefore are more constrained by colder temperatures [37].

Similar to light, temperature varies across multiple scales: most importantly annually, with changes in season, and daily with changes in sunlight. Temperature is therefore not independent from light, as temperatures are consistently higher during daylight hours than at night [39]. However, increased activity when temperatures are extreme can have more severe, and often lethal consequences, relative to the cost of activity at extremes of day and night associated with light [40,41]. This is particularly true for apex predators, who are not at a greater risk of being predated upon at certain times of day. Thus the impact of light on activity must occur within the constraints of that species’ intrinsic thermal limits [39]. This often results in species showing more nocturnal activity during summer months when mean daily temperatures are higher, and more diurnal activity during winter months when temperatures are lower [42].

In addition to the environmental cues which affect activity patterns, biological factors are also important components of animal activity patterns. These biological factors often relate to predator-prey relationships with predators and prey seeking to maximise and minimise overlap in activity respectively [43,44]. In many ways however, humans are the current ‘super predators’ of the planet, and a broad array of species, ranging from prey to predators, all appear to be reacting more strongly to human presence than to each other [45–48]. Human activity is largely diurnal, particularly in rural and natural areas, although human activity also influences the nighttime landscape through artificial light [1,8,10]. A multitude of studies have explored the spatial avoidance of humans by animal species, however only more recently has temporal avoidance been recorded [46,49,50]. Across a range of 62 mammalian species, from 21 orders and 9 families, increased levels of human disturbance have been linked to a shift towards a greater proportion of nocturnal activity with largely unknown impacts on ecosystem level processes [46].

Leopards, though large and charismatic, are notoriously difficult to study given their elusive nature. They are wide ranging and difficult to separate from anthropogenic impacts, often existing in areas where they are regularly exposed to a wide array of threats including poaching and retaliatory killing [51–54]. Leopards are listed as vulnerable by the IUCN Red List due to their declining population size and range, and it is therefore important to understand the full suite of threats this species is facing [55]. Recent literature on the ecological effects of increases in artificial light at night and its effects on interspecific interactions have highlighted light pollution as an emerging threat to animal activity patterns and interspecific interactions. [8,22,23]. However, the relationship between leopard activity and moonlight and whether this relationship changes based on anthropogenic factors remains poorly understood [7,17,18,28,29]. Relatively few studies have looked at the drivers behind leopard activity patterning, particularly in an African context [28,50,56,57]. The relative importance of environmental, ecological and anthropogenic factors on leopard activity may involve complicated interactions. These variables have the potential to invoke additive or opposing effects, increasing the complexity of understanding the drivers behind activity patterning. Here we aim to explore how these covariates influence leopard activity in and around the large, protected area of KNP on both a diel and lunar scale so that their effects on leopard ecology can be better understood.

## Methods

**Fig 1:**
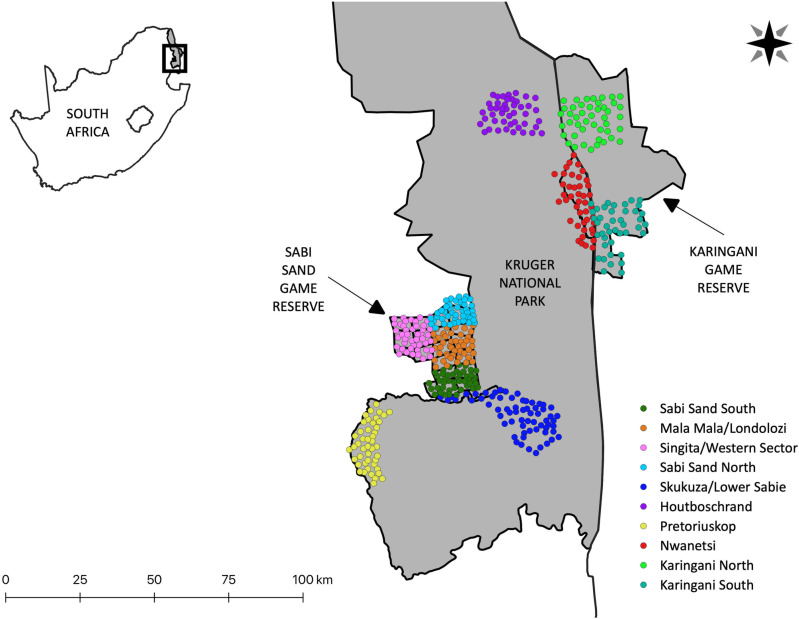
Map of study site. This includes 10 camera trap sites spread between three reserves: Kruger National Park, Sabi Sand Game Reserve and Karingani Game Reserve. Each site consisted of 42-64 paired camera trap stations.

In this study we use data from 10 camera trap sites which collected animal and human activity and relative abundance data across three reserves: Kruger National Park (KNP), Sabi Sand Game Reserve (SSGR) and Karingani Game Reserve (KGR) (Fig 1). All three reserves are formally protected and prohibit consumptive offtake. Between 42 and 64 paired camera trap stations were deployed at each site and remained active for approximately 45 days. Camera traps were attached to trees or metal poles at a heigh of 40 cm above the ground, and stations were set up along roads, drainage lines and game trails to maximize detections. These features are known to provide channels for animal movement in savanna environments where vegetation density can restrict movement on non-established routes [50,58–60]. However, we recognize that this camera placement, optimized for carnivore detection, will not be representative of animal activity across all parts of the environment, and does create a bias against detections of certain prey species that may use alternative movement pathways as a means of predator avoidance. Neighbouring stations were positioned 1.5–3km apart. We used a combination of Panthera V4 and V6 model camera traps which are equipped with a light sensor that activates a white flash when ambient light levels are low, allowing for nocturnal captures.

Capture rate, detected by passive sensors such a as camera traps, has been widely used as a measure of animal activity [18,43,61–63]. We consider captures of the same species as independent if separated by a 30-minute interval as has been done in similar studies of leopard activity [50,56]. We further grouped independent captures into one of six categories: leopards, humans (on foot and in a vehicle), vehicles, pedestrians (humans on foot only), potential prey and potential competitors. Humans on foot were further categorised as vehicle only or pedestrian only captures to investigate whether pedestrians would have a greater impact on leopard activity than humans in vehicles. Potential leopard prey was defined as herbivore species falling within the leopard’s preferred weight range of 10–70 kg and included nine species present in the study area: bushbuck (*Tragelaphus scriptus*), grey duiker (*Sylvicapra grimmia*), grysbok (*Raphicerus sharpei*), impala (*Aepyceros melampus*), klipspringer (*Oreotragus oreotragus*), nyala (*Tragelaphus angasii*), reedbuck (*Redunca arundinum*), steenbok (*Raphicerus campestris*) and suni (*Neotragus moschatus*) [64–66]. We did not obtain enough captures of each species at each site to run species-level analyses, and thus all potential prey species were combined into a single prey category. Similarly, potential leopard competitors include both lions (*Panthera leo*) and spotted hyaenas (*Crocuta crocuta*), and there were insufficient lion captures at each sites to analyse competitor species separately [67,68].

Data analyses were conducted in R version 3.5.2 [69]. We fitted kernel density models to time of day data over a 24-hour period and calculated overall activity estimates for leopards at each site using the ‘activity’ package [70]. We compared temporal overlap in leopard activity patterns between sites as well as between leopard and human, potential prey and potential competitor activity patterns within sites over 24-hour periods using the Dhat 4 overlap index from the ‘overlap’ package [71]. Confidence intervals for Dhat 4 estimates were calculated from 1000 bootstrap samples and were bias corrected.

Day length changes throughout the year at higher latitudes, and the resulting changes in light regimes influence wildlife activity [62]. We thus calculated time of detection relative to sunrise or sunset to control for day length changes. Time of detection for images from 0:00–11:59 was taken relative to sunrise, and time of detection for images from 12:00–23:59 was taken relative to sunset. Times before sunrise or after sunset were represented by a negative integer, while times after sunrise or before sunset were represented by a positive integer. Sunrise and sunset time specific to each station location and detection date were obtained from the R package ‘suncalc’ [72]. We then modelled the relative time of leopard detection in response to a series of explanatory variables representing human, potential prey and potential competitor activity, anthropogenically driven changes to the landscape and seasonal effects. The explanatory variables used were as follows: distance (meters) from each station to the edge of the reserve or nearest human settlement within the reserve (whichever was closer, treated as a single variable and referred to as distance to edge), light pollution level (radiance), relative abundance index (RAI) of all human activity (hereafter referred to as human RAI), RAI of humans in vehicles only (hereafter referred to as vehicle RAI), RAI of humans on foot only (hereafter referred to as pedestrian RAI), potential prey RAI, potential competitor RAI, season, and mean monthly temperature (°Celcius). Light pollution data were obtained for the median month of all surveys (September 2018) from Google Earth Engine’s VIIRS Nighttime Day/Night Band Composites Version 1 dataset and used to provide an indication of level of artificial light at night for all sites over the 1.5 year study [73]. This value represents the mean monthly radiance calculated from nighttime data from the Visible Infrared Imaging Radiometer Suite (VIIRS) Day/Night Band (DNB), measured at a 1km resolution. Human, vehicle, pedestrian, potential prey and potential competitor RAIs were calculated at the level of each camera station for the duration of each survey as the total number of captures relative to the total number of days at least one camera per station was active. Pedestrians and vehicles were included as separate variables because in the study reserves animals are often habituated to the presence of vehicles but they typically flee when exposed to people on foot [74]. Mean monthly temperature (mean temperature per month throughout the whole of KNP averaged over 29 years and provided by KNP’s scientific services [75]), was included based on the month of every detection, as temperature data specific to each location and year were not available. Season was included as a categorical variable, where summer included December, January and February, autumn included March, April and May, winter included June, July and August and spring included September, October and November. Many variables were colinear and thus we ran only univariate models. Models were compared using Akaike’s Information Criterion (AIC) to determine the best predictor of relative time of leopard detection.

To investigate the relationship between leopard movement and lunar light we modelled the RAI of leopards across all stations per calendar day against the fraction of moon illuminated, from the *suncalc* package in R [72]. The fraction of moon illuminated ranges from 0–1, with lower values indicating less moonlight. We grouped RAIs per moon phase, also derived from *suncalc*, (using 11 phases, from 0–10), and used the mean daily leopard RAI from each moon phase in models. We did this for all stations together, and for stations grouped by reserve (SSGR, KNP or KGR) and by site, to look for possible interactions between moon illumination and reserve or site.

Additionally, we investigated whether moon illumination affects hunting success of leopards using observational data collected by guides in the SSGR and generalized linear mixed-effects models. The SSGR, a high end photo-tourism destination, hosts one of the best documented leopard populations given that guides recognise resident leopards by their spot patterns and collect sightings information [68,74,76]. This has allowed for the collection of detailed behavioural data which is usually only accessible through the use of collars. In 2013 Panthera released a data collection software program to all lodges in the reserve, allowing for sightings information to be centralised and for records to be checked against one another to remove duplicates. The reserve has a comprehensive road network and guides traverse the reserve extensively on a daily basis, taking guests on game drives. All guides record the time, date and GPS coordinates of all leopard sightings, identify the individual leopard based on its spots and record behavioural observations including whether the leopard was on a kill, and whether the kill was fresh or not. While guides do not patrol at night, kills made at night are usually observed the following day and recorded as fresh, based on the state of the carcass. Fresh kills are characterised by the presence of bright coloured blood, supple, fresh looking skin and remnants of flesh, and are easy to distinguish in the landscape as having been caught and killed within the previous 24 hours given the lack of carcass decay. While this data does not provide confirmation that the leopard made the kill, leopards are not know to kleptoparasitise kills from other predators as they are more frequently the targets of kleptomarasitism by other species such as lions and spotted hyenas [68]. Since observations were not made at night, we have used observations of a leopard on a fresh kill (< 24 hours old) as representative of a kill that was made at the previous night’s stage of the lunar cycle. We used all sightings records of leopards collected between 2013 and 2019 to run a logistic regression model investigating whether more leopard kills were observed at times of greater lunar illumination. We excluded all observations of leopards on old or kleptoparasitised kills as the level of lunar illumination at the time of the kill was unknown, and though still small, the possibility of an old kill having been kleptoparasitised from another species was higher. These observations were filtered out of our dataset. It is unlikely that this would have biased the data as the likelihood of guides detecting leopards on kills is not affected by moonlight. A total of 5910 observations were filtered out of the dataset due to being of leopards on old or kleptoparasitised kills. In total our remaining dataset consisted of 51597 leopard observations, 7646 of which were of leopards on kills, and the remaining 43951 of which were of leopards not on kills. We did our modelling in a mixed model framework to incorporate individual leopard as a random effect. We modelled whether the leopard in each observation was on a kill or not as a binomial response variable with fraction of the moon illuminated as the explanatory variable.

## Results

**Fig 2:**
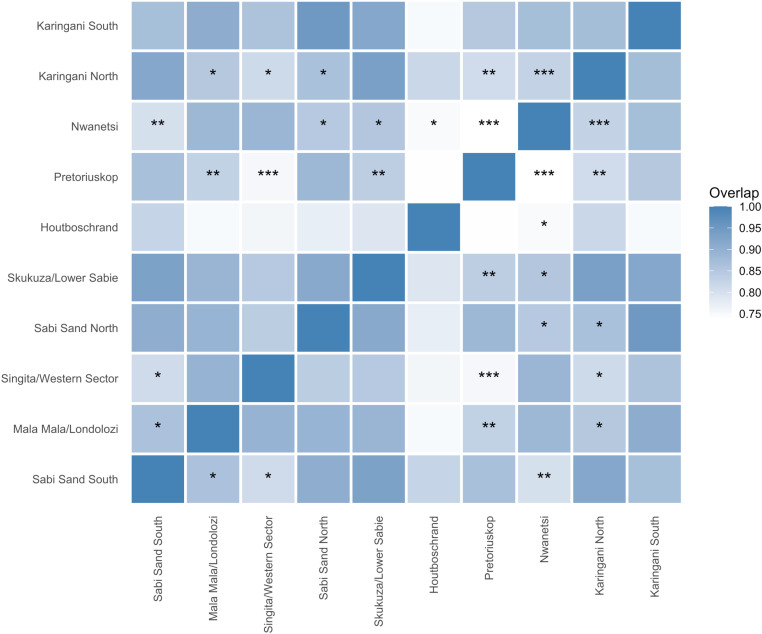
Overlap index in kernel density estimates of leopard activity (Dhat 4) for all combinations of sites. Darker blue represents more overlap in timing of leopard activity. The observed overlap indices were then compared against a null distribution of overlap indices using data sampled randomly with replacement from the combined datasets. Stars represent the level of significance in the difference between observed overlap and randomised overlap distribution (*** = p < 0.001, ** = p < 0.01, * = p < 0.05).

In total we collected 1637 independent captures of leopards over 24 150 trap nights across all 10 sites. The number of independent leopard captures per site ranged from 31 in Houtboschrand to 324 in Sabi Sand South (Table 1). Overall leopard activity estimates were not significantly different between sites (all combinations p > 0.05, exact values in S2 Table) with the proportion of a 24-hour period spent being active ranging from 0.43 ± 0.9 in Houtboschrand to 0.59 ± 0.05/0.06 in Skukuza/Lower Sabie and Mala Mala Londolozi respectively (S1 Table), however significant differences in timing of activity were present for certain combinations of sites (Fig 2).

**Table 1:** Summary of data collected from camera trap surveys. The number of trap nights refers to the cumulative number of nights over the course of the survey that at least one camera per station was active. The number of captures refers to the number of independent captures, using a threshold of 30 minutes between successive captures of the same species or target group. Pedestrian captures refer to humans on foot, and human captures refer to both vehicles and humans on foot.

Reserve	Site	Year	Number of stations	Trap nights	Number of leopard captures	Number of pedestrian captures	Number of vehicle captures	Number of human captures	Number of prey captures	Number of competitor captures
SSGR	Sabi Sand South	2018	45	2656	324	794	6428	6660	2074	812
SSGR	Mala Mala/Londolozi	2018	46	2299	196	567	3367	3588	2055	772
SSGR	Singita/Western Sector	2018	45	2317	90	743	7003	7193	2936	777
SSGR	Sabi Sand North	2019	43	2168	185	844	5155	5485	2261	770
KNP	Skukuza/Lower Sabie	2018	64	4064	275	734	2090	2608	2331	1126
KNP	Houtboschrand	2018	45	2252	31	391	2439	2666	1351	534
KNP	Pretoriuskop	2018	45	2134	155	535	1355	1735	1305	508
KNP	Nwanetsi	2018	43	2113	103	1624	1079	2152	1594	777
KGR	Karingani North	2019	45	2186	217	279	1791	1928	993	217
KGR	Karingani South	2019	42	1961	61	672	2566	2904	1002	132

**Fig 3:**
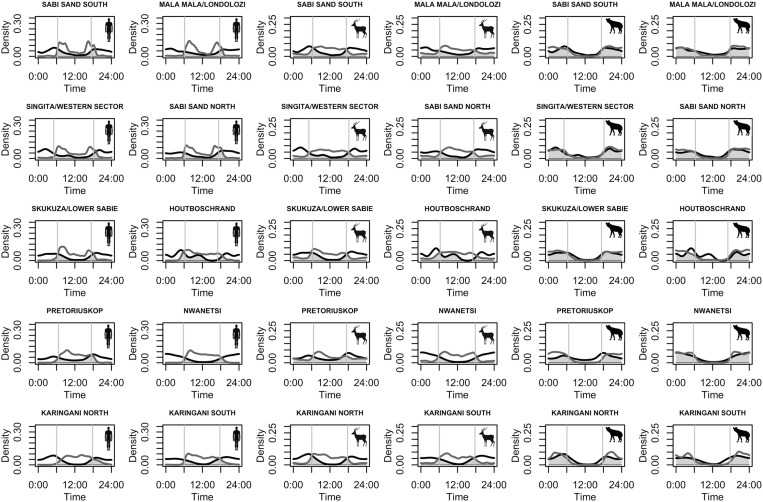
Kernel density models. These show temporal overlap between the activity of leopard (black) and humans, potential prey species and potential competitor species (grey). Pale grey vertical lines show times of sunrise and sunset.

Kernel density models of activity patterns over a 24-hour period indicate that leopard activity was predominantly nocturnal at all sites (Fig 3). Variation between sites was apparent with leopards showing crepuscular peaks in activity at certain sites and consistent nocturnal activity with no sunrise or sunset peaks at other sites. Despite these differences, activity levels were always lowest in the middle of the day at all sites. The overlap in leopard activity patterning between sites measured through the Dhat 4 index was generally high, ranging from 0.74 (Pretoriuskop vs Nwanetsi) to 0.95 (Sabi Sand North vs Karingani South) (Fig 2). Fifteen site combinations showed statistically different leopard activity patterns (Fig 2).

There is evidence of temporal partitioning between leopard and human activity with leopards being predominantly nocturnal and humans predominantly diurnal at all sites (Fig 3). Human activity usually peaked around sunrise, showed another peak around sunset and then virtually ceased overnight across all sites in accordance with the rules restricting nighttime tourist movement within the reserves. Leopard activity began to taper off before sunrise but was not at its lowest point at the times that humans were most active. Dhat 4 overlap estimates ranged from 0.24 at Nwanetsi to 0.49 at Pretoriuskop (S3 Table). Potential prey showed predominantly diurnal activity, with slight peaks in activity in the two hours following sunrise at some sites (Fig 3). Activity levels remained relatively consistent throughout the day at all sites and then decreased around sunset, remaining low throughout the night compared to daytime activity levels. Overlap between leopard and potential prey activity was higher than for leopard and human activity, with Dhat 4 estimates ranging from 0.38 at Nwanetsi to 0.73 at Pretoriuskop (S3 Table). Potential competitors showed similar activity patterns to leopards with activity being almost entirely nocturnal and characterised by crepuscular peaks, though with even less diurnal activity than leopards (Fig 3). Dhat 4 overlap estimates show a high proportion of overlap in activity between leopards and their potential competitors, ranging from 0.69 at Pretoriuskop to 0.88 at Nwanetsi and Skukuza/Lower Sabie (S3 Table).

**Fig 4:**
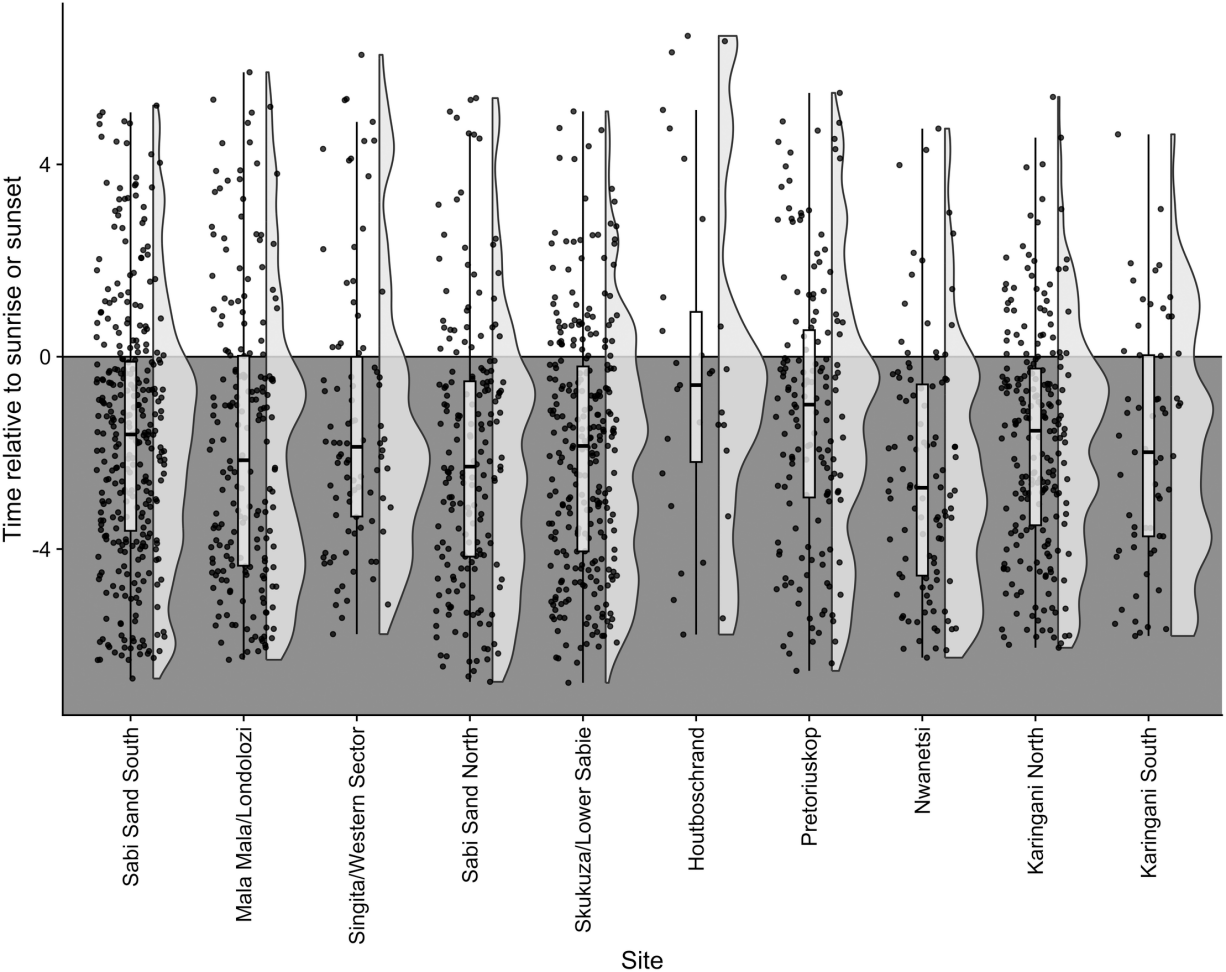
Time of detection relative to sunrise or sunset for leopards across 10 sites in and around Kruger National Park. Time of detection for images from 0:00-11:59 was taken relative to sunrise, and time of detection for images from 12:00-23:59 was taken relative to sunset. The lower, shaded area represents images captured at night, and the upper, white area represents images captured in the daytime. Boxplots represent the mean and standard deviation, and raincloud plots, shown in light grey, illustrate the intensity and spread of data points.

When leopard captures are scored relative to sunrise or sunset, whichever is nearest to the capture, the mean time of capture for all sites lies between 0.5 and 3 hours before sunrise or after sunset and is closest to 2 hours for most sites (Fig 4). Relative time of capture was closest to sunrise or sunset at Houtboschrand, and furthest from sunrise or sunset at Nwanetsi. Linear regression and AIC based model selection indicates that the model best able to predict relative time of leopard capture includes pedestrian RAI as a covariate, and the next best model includes human RAI as a covariate, followed by vehicle RAI (Table 2). AIC scores between the first and second models differ by only 0.58 indicating that there is almost as much support for the impact of any human presence (in a vehicle or on foot) as there is for exclusively pedestrians on the relative time of capture of leopards. Vehicle RAI show less support, but still more than the null model with a delta AIC of 1.91. Pedestrian RAI, Human RAI and Vehicle RAI all show significant negative relationships with time of leopard capture meaning that higher RAIs result in more nocturnal captures (estimate: -0.173, t: -2.585, p-value: < 0.05 and estimate: -0.166, t: -2.470, p-value: < 0.05 and estimate: 0.146, t: -2.184, p-value: < 0.05 respectively). None of the other models include variables with significant impacts on the timing of leopard captures on camera traps.

**Table 2:** Model selection table investigating variables that could impact the timing of leopard activity. Time refers to time of detection relative to sunrise or sunset for leopards across 10 sites in and around Kruger National Park. Time of detection for images from 0:00-11:59 was taken relative to sunrise, and time of detection for images from 12:00-23:59 was taken relative to sunset. Time ~ 1 refers to the null model.

Model	Number of Parameters	AIC	Delta AIC	AIC Weight
Time ~ Pedestrian RAI	3	7913.31	0	0.41
Time ~ Human RAI	3	7913.89	0.58	0.31
Time ~ Vehicle RAI	3	7915.22	1.91	0.16
Time ~ 1	2	7917.99	4.68	0.04
Time ~ Competitor RAI	3	7919.15	5.84	0.02
Time ~ Light pollution	3	7919.9	6.59	0.02
Time ~ Distance to edge	3	7919.93	6.62	0.01
Time ~ Temperature	3	7919.95	6.64	0.01
Time ~ Prey RAI	3	7919.95	6.64	0.01
Time ~ Season	5	7921.58	8.27	0.01

**Fig 5:**
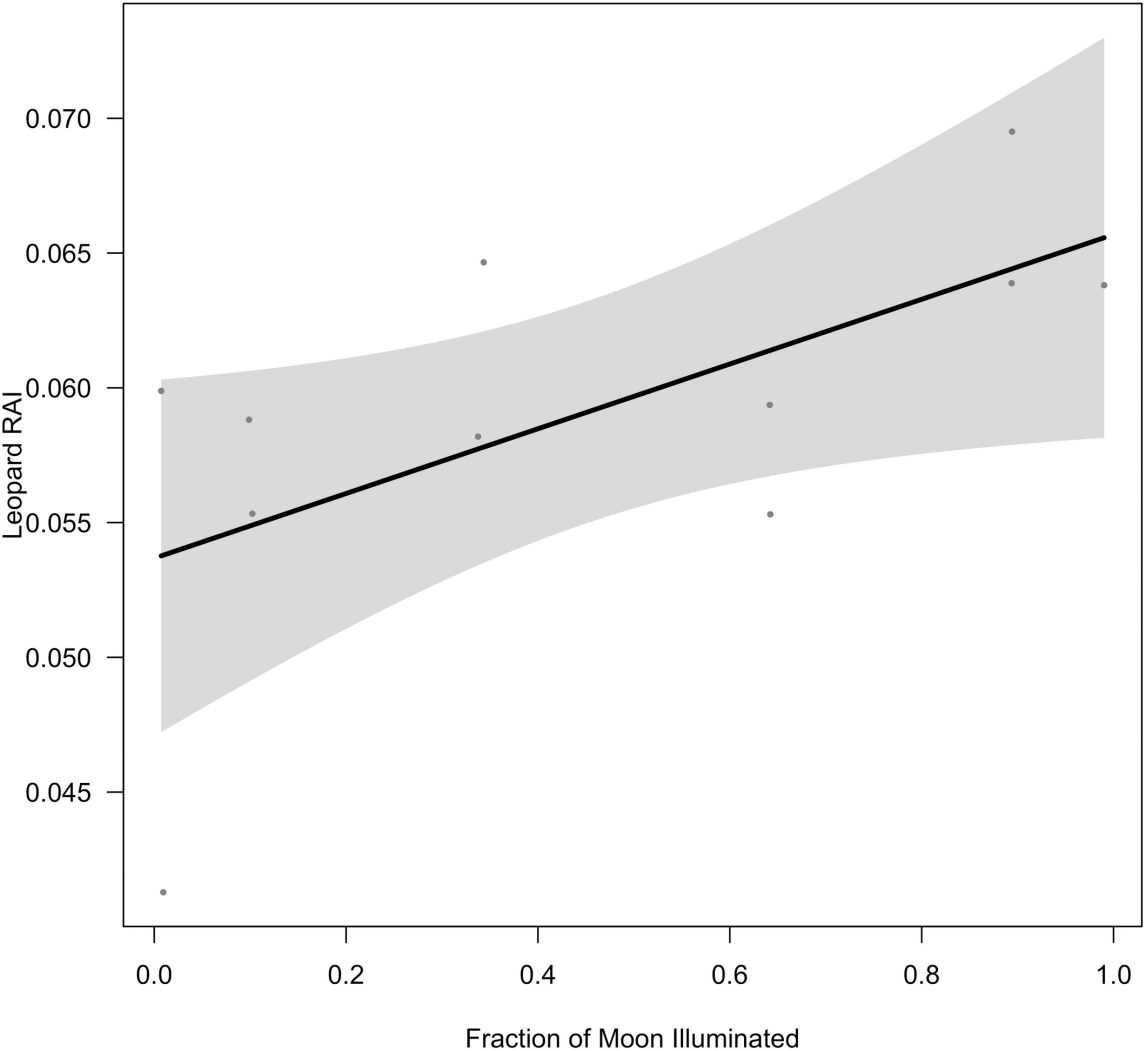
Relationship between moon illumination and leopard RAI. Estimate = 0.012, Standard Error = 0.005, t-value = 2.371, P < 0.05.

Moonlight had a significant effect on leopard activity levels. Increased moon illumination positively correlated with higher leopard RAI scores per night (Estimate = 0.012, Standard Error = 0.005, t-value = 2.371, P < 0.05) (Fig 5). There was however no interaction between fraction of the moon illuminated and reserve (SSGR, KNP or KGR), or between fraction of the moon illuminated and site (S4 Table). Leopard-kill success also appears to be affected by moonlight, with fraction of the moon illuminated having a negative effect on whether leopard observations recorded during daylight hours of the following day involved a kill (Estimate = -0.105, Standard Error = 0.037, z-value = -2.823, p < 0.005).

## Discussion

Animal activity patterns are driven by a complex suite of potentially interacting factors, which can be difficult to identify and measure [43,46,56,62,77,78]. Changing activity patterns in response to human impacts have only been recorded relatively recently, and are considered crucial for many species to both coexist alongside humans, and tolerate associated environmental changes [46,63,79]. There are three major ways in which animal activity patterns can change in response to humans: overall levels of activity can change, the location of activity can change and the timing of activity can change [13,80]. In this study we have assessed overall leopard activity levels, timing of leopard activity on a daily scale and the drivers behind changes in the timing of activity. Additionally, we explored leopard activity across the lunar cycle, and how this correlates with kill success.

Overall activity levels provide a high-level indication of animal activity, but miss the nuances of how activity is spread across a 24 hour period. This is reflected in the results of this study as we found no differences in overall level of leopard activity between sites, but significant differences in the timing of leopard activity. The predominantly nocturnal behaviour of leopards in all sites surveyed throughout this study is to be expected given that leopard hunting success was found to be higher at night than during the day in the Cederberg Mountains of South Africa [31], leopards were found to cover greater distance at night as opposed to during the day in the Kenyan rangelands [28], and leopard activity has been characterised as largely nocturnal in parts of Tanzania and South Africa [50,57]. Leopards are camouflaged ambush hunters, relying on stealth and crypsis for hunting success [64]. Most small and medium ungulate species (a major source of leopard prey) rely predominantly on vision for predator detection, and their vision is substantially less effective at night [24,27]. Leopards however have excellent night vision, allowing them to see prey at low levels of illumination, leading to more successful hunts at night [31,81]. Crepuscular peaks in leopard activity may be linked to increased prey encounters at these times, with prey more active around sunrise and sunset [24,56], or to increased prey catchability at dusk and dawn driven by a trade-off between the advantages accrued by predators of approaching prey with limited light availability and ambushing prey with some light still present [82].

Unlike leopards, humans are predominantly diurnal and this is reflected in a shift towards greater nocturnality in many wild animal species living in human dominated environments [45–47,56,77,78]. The diurnal nature of humans is exaggerated in this study by regulations limiting human movement after dark due to anti-poaching efforts. These regulations are particularly stringent in KNP. Results from this study indicate that of the covariates investigated, pedestrian activity has the strongest negative impact on the timing of leopard activity, followed closely by general human activity, consisting of a combination of both pedestrians and vehicles. Vehicle activity had less of an impact, though still significant, on the timing of leopard activity than the activity of humans of foot. These results support the ubiquity of human disturbance as a driver of altered wildlife activity patterning globally [46]. The significant negative relationships between pedestrian RAI and timing of leopard activity, human RAI and timing of leopard activity and vehicle RAI and timing of leopard activity indicates that mean timing of leopard capture is likely to occur deeper into the nighttime hours relative to sunrise or sunset when pedestrian, human or vehicle RAI is greater. Model selection indicates that pedestrian RAI better explains differences in timing of leopard activity than human RAI or vehicle RAI. This illustrates that humans on foot have a stronger influence on timing of leopard activity than humans in vehicles. Similar results were obtained using data from collared leopards which revealed changes in habitat selection corresponded to changes in human activity, indicating that leopards avoided areas where they were likely to encounter humans in the daytime, and certain individuals selected for those same areas at nighttime [28].

The definition of human disturbance in studies exploring the impacts of anthropogenic activity on wildlife is however, not straightforward. While human RAI estimated from camera traps has been widely used as an indicator of human disturbance, it does not differentiate between different types of human activity, which can impact the degree to which human activity affects animal populations [77,83,84]. Human RAI is not a suitable metric for consumptive human activities in this study. Given that hunting is illegal in these reserves, any form of illegal offtake occurs by poachers who avoid travelling on roads, drainage lines and game trails to minimise their chances of being caught by rangers. Poachers therefore remain largely undetected by these survey methods and cannot be reliably quantified in this dataset. Anecdotal knowledge of the study area points towards KGR as having the most potential for illegal offtake around the time of the study given its proximity to impoverished communities combined with its status as a recently developed protected area with fences that were still incomplete at the time of camera trapping. The SSGR on the other hand, despite having high levels of human activity, has a substantial security budget that allows for well maintained and monitored fence lines and a high density of anti-poaching units, making illegal human offtake highly unlikely [60]. KNP is intermediate with regards to the potential for consumptive human activities. Trends in total human activity and consumptive human activity between reserves are therefore substantially different and could be masking the effects of one another.

Despite the potentially confounding and unaccounted for variable of consumptive human offtake, the effects of overall human activity remain strong enough to significantly affect leopard activity patterns in these formally protected areas where human activity is predominantly non-consumptive, with no consumptive activities legally permitted. These findings are supported by a global meta-analysis which found that increased nocturnality was observed across a wide range of species, regardless of the inherent activity patterns of the animal species in question (diurnal or nocturnal), and regardless of the type of human activity conducted (consumptive, non-consumptive or simply the presence of infrastructure) [46]. Given that most human activity in these reserves in non-consumptive and therefore not directly harmful to the leopards themselves, leopards appear to have become somewhat accustomed to the presence of humans in vehicles, seen through the higher AIC scores of the models including vehicle only activity and all human activity compared to pedestrian only activity. This has been recorded anecdotally in the SSGR, where leopards appear unbothered by the presence of vehicles, allowing particularly good viewing opportunities for passengers [74].

Despite the important role that human activity has on leopard activity patterns in the greater Kruger region of South Africa, distance to areas of greater human activity (either the edge of the reserve or a human settlement within the reserve, whichever was closer) is not a significant predictor of timing of leopard activity. This indicates that the relative activity of humans within the study area was more important than the proximity of a station to areas of higher overall human activity outside the study area. Human activity is intricately linked to a number of other forms of disturbance, such as sensory pollution in the form of light and noise [11]. The individual effects of each of these can be difficult to disentangle, but light pollution alone does not appear to be a predictor of leopard activity patterning in this study. Light pollution and distance to human settlements or the reserve edge are fundamentally linked, as artificial light at night is emitted by human settlements. While on a global scale light pollution has been directly linked to changes in the timing of animal activity, it is likely that there was not sufficient variation in this variable across the study site for meaningful changes in timing of activity to be observed. Had we placed camera traps in the land surrounding these reserves, where light pollution is stronger, the effect of artificial light at night on leopard activity may have been more pronounced.

Interestingly, neither potential prey nor potential competitor RAIs appear to impact the relative timing of leopard activity. Leopards are known to feed primarily on small to medium sized ungulates, most of which are diurnal given their reliance on vision for vigilance [64–66]. Despite being less active at night these prey species are nevertheless still vulnerable to leopards who benefit from improved crypsis in low light conditions [31]. Temporal partitioning between competitive predatory species has been widely observed, with subordinate predators such as cheetahs and wild dogs generally showing a shift towards more diurnal behaviour to avoid overlapping with more dominant nocturnal predators such as lions and spotted hyaenas [3,17,85]. Leopards fall somewhere in the middle of this spectrum, being dominant over wild dogs and cheetahs but subordinate to spotted hyaenas who kleptoparasitise kills and lions who account for a significant proportion of leopard mortality in some areas [3,17,67,68]. Therefore, in general, leopards are assumed to shift towards more nocturnal behaviour in response to human disturbance, and more diurnal behaviour in response to competition with other predators [31,86]. A lack of effect of competitor activity on leopard activity, as was the case at these sites, has been observed before [67]. Unless lion densities are particularly high [51], lion distribution does not affect the spatial distribution of leopard activity, as the spatial avoidance of lions by leopards appears to happen at a much smaller scale, with leopards retreating to elevated, less accessible locations such as tree branches to avoid contact when in close proximity to lions (<50m) [67]. While hyenas have been found to show high levels of spatiotemporal overlap with leopards [60,87,88] it has also been observed that leopards avoid hyena kleptoparasitism by caching prey in trees which are inaccessible to hyaenas [68]. It is therefore likely that their arboreal habits allow leopards to avoid both spatial and temporal niche partitioning with competitors. These fine scale behaviours which help to avoid negative encounters with competitors such as lions and hyenas without requiring spatial avoidance or temporal partitioning over the diel cycle do not appear to be sufficient for leopards to avoid the perceived threat of the human super predator, resulting in a shift towards more nocturnal activity in areas of higher human activity.

All animal activity is limited by the constraints of environmental variables such as temperature [4,56,61,62], which varies seasonally. Mean minimum and maximum temperatures across the study sites vary substantially between seasons, with minimum daily temperatures ranging from 9.5°C in winter to 20.4°C in summer, and maximum daily temperatures ranging from 26.1°C in winter to 32.2°C in summer. However, neither mean monthly temperature nor season show any impact on timing of leopard activity in this study. This indicates that in this region leopards appear to shift their activity patterns according to anthropogenic stressors without constraints imposed by the limits of their thermal tolerance. Effects of seasonal temperature variation on activity patterning has been observed in snow leopards (*Panthera uncia*) who display a greater proportion of diurnal activity in winter months, presumably for thermoregulatory reasons [82]. However, no studies appear to have documented any effects of temperature on leopard activity patterning in Africa.

The timing of leopard activity, while variable on a daily scale, is also variable on a lunar scale, seen through the positive correlation between increased lunar illumination and leopard activity. The increased likelihood of observing leopards on a kill during stages of the lunar cycle with lower levels of lunar illumination provides a potential explanation for this. Increased leopard activity during periods of brighter moonlight could indicate that it takes more activity, and consequently energy, for leopards to meet their feeding requirements because hunting success is lower at higher light levels. This theory is supported by evidence of leopards making more kills during times of lower lunar illumination in the Cederberg Mountains of the Western Cape of South Africa, and by data indicating that prey vulnerability increases with darkness [24,31]. If moonlight affects leopard activity and kill success over the lunar scale, as these results show, and artificial light at night is increasing globally by 6% annually, then it is unlikely that this rise in artificial light at night has not already impacted leopard activity levels to some extent [89].

Overall, in the greater Kruger ecosystem, pedestrian RAI is the most important variable influencing diel leopard activity patterns with increased nighttime activity in areas with higher pedestrian activity. These findings support the overwhelming global bank of evidence that human disturbance influences animal activity, even where consumptive offtake is not permitted. Expanding camera trap surveys into mixed land uses outside of protected areas may offer more insights into the relative importance of consumptive versus non-consumptive human activity on activity patterns, and other variables associated with human activity such as light pollution. This is particularly important given our finding that leopard hunting success varies according to the lunar cycle, indicating that hunting success is impacted by nighttime illumination. Evidence of the impact of human activity on the activity patterns of predator species such as the leopard cautions that even in formally protected areas such as Kruger National Park ecosystem processes are not independent from anthropogenic disturbance.

## Supporting information

S1 Table Leopard activity estimates derived from kernel density models. These represent the proportion of the day spent active for leopards at each of the 10 study sites. A Wald test shows no significant differences in overall activity estimates between any of the sites (p > 0.05 for all combinations of sites).
(PDF)

S2 Table Comparisons between leopard activity estimates for all sessions. These are derived from kernel density estimates and include associated Wald statistics and p values.
(PDF)

S3 Table Estimates of activity pattern overlap between leopards and humans, prey and competitors based on time of observation. Prey includes bushbuck, grey duiker, grysbok, impala, klipspringer, nyala, reedbuck, steenbok and suni and competitors include lions and hyaenas. Confidence intervals (CI) are calculated from 1000 bootstrap samples and have been bias corrected.
(PDF)

S4 Table Model outputs from regression models looking at the relationship between the fraction of the moon illuminated and leopard RAI per night. Leopard RAI was calculated per night for all stations from all sites combined. Models 2 and 3 investigate whether there is an interaction between the fraction of the moon illuminated and the reserve (SSGR, KNP or KGR, with KGR as the baseline), or the site (with Houtboschrand as the baseline).
(PDF)

S5 File Activity input data. (CSV)

S6 File Leopard kill observation data. (CSV)
