# Supplementary material for: Dinner in the dark: Factors influencing leopard activity patterns within a large protected area
Source: PLoS One. 2025 May 22;20(5):e0324329. doi: 10.1371/journal.pone.0324329 (PMC12097597; doi:10.1371/journal.pone.0324329)
Supplement: S1 Table — These represent the proportion of the day spent active for leopards at each of the 10 study sites. A Wald test shows no significant differences in overall activity estimates between any of the sites (p > 0.05 for all combinations of sites). (PDF) [file pone.0324329.s001.pdf]

S1 Table: Leopard activity estimates derived from kernel density models. These represent the proportion of the day spent active for leopards at each of the 10 study sites. A Wald test shows no significant differences in overall activity estimates between any of the sites ($p > 0.05$ for all combinations of sites).

Site	Activity Estimate	Standard Error
Sabi Sand South	0.53	0.05
Mala Mala/Londolozi	0.59	0.06
Singita/Western Sector	0.48	0.07
Sabi Sand North	0.58	0.06
Skukuza/Lower Sabie	0.59	0.05
Houtboschrand	0.43	0.09
Pretoriuskop	0.54	0.07
Nwanetsi	0.52	0.05
Karingani North	0.49	0.05
Karingani South	0.58	0.08
