# Supplementary material for: Dinner in the dark: Factors influencing leopard activity patterns within a large protected area
Source: PLoS One. 2025 May 22;20(5):e0324329. doi: 10.1371/journal.pone.0324329 (PMC12097597; doi:10.1371/journal.pone.0324329)
Supplement: S2 Table — These are derived from kernel density estimates and include associated Wald statistics and p values. (PDF) [file pone.0324329.s002.pdf]

S2 Table: Comparisons between leopard activity estimates for all sessions. These are derived from kernel density estimates and include associated Wald statistics and p values.

Sessions	Difference in Activity	Standard Error	Wald Statistic	p-value
Sabi Sand South: Mala Mala/Londolozi	-0.061	0.076	0.642	0.423
Sabi Sand South: Singita/Western Sector	0.046	0.081	0.313	0.576
Sabi Sand South: Sabi Sand North	-0.050	0.075	0.444	0.505
Sabi Sand South: Skukuza/Lower Sabie	-0.061	0.070	0.766	0.381
Sabi Sand South: Houtboschrand	0.098	0.104	0.876	0.349
Sabi Sand South: Pretoriuskop	-0.014	0.086	0.025	0.874
Sabi Sand South: Nwanetsi	0.013	0.069	0.035	0.852
Sabi Sand South: Karingani North	0.040	0.070	0.326	0.568
Sabi Sand South: Karingani South	-0.054	0.089	0.369	0.544
Mala Mala/Londolozi: Singita/Western Sector	0.106	0.088	1.445	0.229
Mala Mala/Londolozi: Sabi Sand North	0.011	0.082	0.018	0.893
Mala Mala/Londolozi: Skukuza/Lower Sabie	0.000	0.078	0.000	0.998
Mala Mala/Londolozi: Houtboschrand	0.158	0.110	2.076	0.150
Mala Mala/Londolozi: Pretoriuskop	0.047	0.093	0.258	0.611
Mala Mala/Londolozi: Nwanetsi	0.074	0.077	0.916	0.338
Mala Mala/Londolozi: Karingani North	0.101	0.078	1.656	0.198
Mala Mala/Londolozi: Karingani South	0.006	0.096	0.005	0.946
Singita/Western Sector: Sabi Sand North	-0.095	0.087	1.188	0.276
Singita/Western Sector: Skukuza/Lower Sabie	-0.106	0.083	1.639	0.200
Singita/Western Sector: Houtboschrand	0.052	0.114	0.209	0.647
Singita/Western Sector: Pretoriuskop	-0.059	0.097	0.370	0.543
Singita/Western Sector: Nwanetsi	-0.033	0.082	0.158	0.691
Singita/Western Sector: Karingani North	-0.005	0.084	0.004	0.949
Singita/Western Sector: Karingani South	-0.100	0.100	0.991	0.320
Sabi Sand North: Skukuza/Lower Sabie	-0.011	0.076	0.022	0.883
Sabi Sand North: Houtboschrand	0.147	0.109	1.824	0.177
Sabi Sand North: Pretoriuskop	0.036	0.092	0.154	0.695
Sabi Sand North: Nwanetsi	0.062	0.076	0.683	0.409
Sabi Sand North: Karingani North	0.090	0.077	1.354	0.245
Sabi Sand North: Karingani South	-0.005	0.095	0.002	0.961
Skukuza/Lower Sabie: Houtboschrand	0.159	0.106	2.248	0.134
Skukuza/Lower Sabie: Pretoriuskop	0.047	0.088	0.290	0.590
Skukuza/Lower Sabie: Nwanetsi	0.074	0.071	1.087	0.297
Skukuza/Lower Sabie: Karingani North	0.101	0.072	1.947	0.163
Skukuza/Lower Sabie: Karingani South	0.007	0.091	0.005	0.942
Houtboschrand: Pretoriuskop	-0.111	0.117	0.901	0.343
Houtboschrand: Nwanetsi	-0.085	0.105	0.652	0.419
Houtboschrand: Karingani North	-0.057	0.106	0.293	0.589
Houtboschrand: Karingani South	-0.152	0.120	1.611	0.204
Pretoriuskop: Nwanetsi	0.026	0.087	0.092	0.761
Pretoriuskop: Karingani North	0.054	0.088	0.371	0.543
Pretoriuskop: Karingani South	-0.041	0.104	0.152	0.696
Nwanetsi: Karingani North	0.027	0.071	0.147	0.702
Nwanetsi: Karingani South	-0.067	0.090	0.553	0.457
Karingani North: Karingani South	-0.094	0.092	1.063	0.302
