# Supplementary material for: Dinner in the dark: Factors influencing leopard activity patterns within a large protected area
Source: PLoS One. 2025 May 22;20(5):e0324329. doi: 10.1371/journal.pone.0324329 (PMC12097597; doi:10.1371/journal.pone.0324329)
Supplement: S3 Table — Prey includes bushbuck, grey duiker, grysbok, impala, klipspringer, nyala, reedbuck, steenbok and suni and competitors include lions and hyaenas. Confidence intervals (CI) are calculated from 1000 bootstrap samples and have been bias corrected. (PDF) [file pone.0324329.s003.pdf]

S3 Table: Estimates of activity pattern overlap between leopards and humans, prey and competitors based on time of observation. Prey includes bushbuck, grey duiker, grysbok, impala, klipspringer, nyala, reedbuck, steenbok and suni and competitors include lions and hyaenas. Confidence intervals (CI) are calculated from 1000 bootstrap samples and have been bias corrected.

Site	Human Overlap Estimate (Dhat 4)	Human Bootstrap 95% CI	Prey Overlap Estimate (Dhat 4)	Prey Bootstrap 95% CI	Competitor Overlap Estimate (Dhat 4)	Competitor Bootstrap 95% CI
Sabi Sand South	0.42	0.36 – 0.45	0.54	0.47 – 0.56	0.82	0.77 – 0.87
Mala Mala/Londolozi	0.42	0.34 – 0.45	0.59	0.51 – 0.63	0.87	0.83 – 0.94
Singita/Western Sector	0.39	0.27 – 0.44	0.48	0.36 – 0.54	0.87	0.82 – 0.97
Sabi Sand North	0.45	0.37 – 0.49	0.51	0.42 – 0.54	0.86	0.82 – 0.94
Skukuza/Lower Sabie	0.40	0.33 – 0.43	0.55	0.48 – 0.58	0.88	0.84 – 0.94
Houtboschrand	0.47	0.30 – 0.59	0.56	0.41 – 0.67	0.71	0.57 – 0.87
Pretoriuskop	0.49	0.41 – 0.55	0.73	0.65 – 0.77	0.69	0.62 – 0.76
Nwanetsi	0.24	0.13 – 0.26	0.38	0.27 – 0.43	0.88	0.86 – 0.98
Karingani North	0.38	0.30 – 0.40	0.46	0.37 – 0.49	0.78	0.71 – 0.84
Karingani South	0.40	0.28 – 0.48	0.49	0.38 – 0.57	0.78	0.70 – 0.91
