# Supplementary material for: Dinner in the dark: Factors influencing leopard activity patterns within a large protected area
Source: PLoS One. 2025 May 22;20(5):e0324329. doi: 10.1371/journal.pone.0324329 (PMC12097597; doi:10.1371/journal.pone.0324329)
Supplement: S4 Table — Leopard RAI was calculated per night for all stations from all sites combined. Models 2 and 3 investigate whether there is an interaction between the fraction of the moon illuminated and the reserve (SSGR, KNP or KGR, with KGR as the baseline), or the site (with Houtboschrand as the baseline). (PDF) [file pone.0324329.s004.pdf]

S4 Table: Model outputs from regression models looking at the relationship between the fraction of the moon illuminated and leopard RAI per night. Leopard RAI was calculated per night for all stations from all sites combined. Models 2 and 3 investigate whether there is an interaction between the fraction of the moon illuminated and the reserve (SSGR, KNP or KGR, with KGR as the baseline), or the site (with Houtboschrand as the baseline).

Coefficient	Estimate	Standard Error	t	p-value	Significance
		Model 1: RAI ~ Illumination			
(Intercept)	0.054	0.003	18.386	0	***
Illumination	0.012	0.005	2.371	0.042	*
		Model 2: RAI ~ Illumination * Reserve			
(Intercept)	0.04	0.006	7.219	0	***
Illumination	0.029	0.01	3	0.006	**
ReserveKNP	0.003	0.008	0.397	0.694	
ReserveSSGR	0.031	0.008	3.897	0.001	***
Illumination:ReserveKNP	-0.02	0.014	-1.461	0.156	
Illumination:ReserveSSGR	-0.022	0.014	-1.631	0.115	
		Model 3: RAI ~ Illumination * Site			
(Intercept)	0.008	0.009	0.833	0.407	
Illumination	0.010	0.016	0.631	0.529	
SiteKaringani_North	0.057	0.013	4.285	0	***
SiteKaringani_South	0.013	0.013	0.998	0.321	
SiteMala_Mala/Londolozi	0.063	0.013	4.761	0	***
SiteNwanetsi	0.033	0.013	2.495	0.014	*
SitePretoriuskop	0.047	0.013	3.536	0.001	***
SiteSabi_Sand_South	0.098	0.013	7.323	0	***
SiteSabi_Sand_North	0.069	0.013	5.191	0	***
SiteSingita/Western_Sector	0.016	0.013	1.217	0.227	
SiteSkukuza/Lower_Sabie	0.059	0.013	4.4	0	***
Illumination:SiteKaringani_North	0.028	0.023	1.231	0.221	
Illumination:SiteKaringani_South	-0.001	0.023	-0.035	0.972	
Illumination:SiteMala_Mala/Londolozi	-0.008	0.023	-0.362	0.718	
Illumination:SiteNwanetsi	-0.002	0.023	-0.103	0.918	
Illumination:SitePretoriuskop	0.005	0.023	0.232	0.817	
Illumination:SiteSabi_Sand_South	0.000	0.023	-0.015	0.988	
Illumination:SiteSabi_Sand_North	-0.009	0.023	-0.371	0.711	
Illumination:SiteSingita/Western_Sector	0.008	0.023	0.352	0.725	
Illumination:SiteSkukuza/Lower_Sabie	-0.018	0.023	-0.764	0.447	
